# System analysis based on the lysosome-related genes identifies HPS4 as a novel therapy target for liver hepatocellular carcinoma

**DOI:** 10.3389/fonc.2023.1221498

**Published:** 2023-09-13

**Authors:** Ke‐Jie He, Zhiqiang Nie

**Affiliations:** ^1^ The Quzhou Affiliated Hospital of Wenzhou Medical University, Quzhou People’s Hospital, Quzhou, Zhejiang, China; ^2^ Global Health Research Center, Guangdong Cardiovascular Institute, Guangdong Provincial People’s Hospital, Guangdong Academy of Medical Sciences, Southern Medical University, Guangzhou, China

**Keywords:** LIHC, HPS4, gene mutation, copy number variation, methylation, immune, prognostic signature, chemotherapy response

## Abstract

**Background:**

Liver cancer is a leading cause of cancer-related deaths worldwide. Lysosomal dysfunction is implicated in cancer progression; however, prognostic prediction models based on lysosome-related genes (LRGs) are lacking in liver cancer. This study aimed to establish an LRG-based model to improve prognosis prediction and explore potential therapeutic targets in liver cancer.

**Methods:**

Expression profiles of 61 LRGs were analyzed in The Cancer Genome Atlas liver cancer cohorts. There were 14 LRGs identified, and their association with clinical outcomes was evaluated. Unsupervised clustering, Cox regression, and functional assays were performed.

**Results:**

Patients were classified into high-risk and low-risk subgroups based on the 14 LRGs. The high-risk group had significantly worse overall survival. Aberrant immune infiltration and checkpoint expression were observed in the high-risk group. Furthermore, HPS4 was identified as an independent prognostic indicator. Knockdown of HPS4 suppressed liver cancer cell proliferation and induced apoptosis.

**Conclusion:**

This study developed an LRG-based prognostic model to improve risk stratification in liver cancer. The potential value of HPS4 as a therapeutic target and biomarker was demonstrated. Regulation of HPS4 may offer novel strategies for precision treatment in liver cancer patients.

## 1 Introduction

Globally, LIHC ranks sixth among the most common cancers (1, 2). There are projected to be more than one million cases of liver cancer by 2030 (3). The number of patients who die from LIHC each year exceeds half a million (4). In the early stages, LIHC has no symptoms but rapidly progresses (5). At present, LIHC is mainly treated with liver transplantation, hepatic resection, and medication (6). LIHC can be surgically excised in only 10%–20% of patients, but recurrences are common (7). The current drug therapy has limited efficacy for LIHC because it is chemoresistant (8, 9). Despite the application of new treatment strategies for LIHC, efficacy remains unsatisfactory (10). Thus, identification of specific prognostic markers is essential for guiding LIHC therapy and improving OS of patients.

In all types of cells, lysosomes are membrane-bound phospholipid bilayers that catabolize protein degradation and recycle it through phagocytosis, endocytosis, and autophagy (11–13). It has been reported that lysosome functions are drastically altered during cancer progression, including alterations in lysosome volume, localization, and composition within the cell (14, 15). In addition, lysosomes have been found to be routed to the periphery of the cell under multiple stimuli that can result in a variety of pathological conditions (11). In malignant transformations, lysosomes are juxtaposed to the plasma membrane, which contributes to cell invasion and migration (16, 17). A number of cancers show significant overexpression of lysosomal hydrolases, which increases invasion of tumor. TMEM106B has been reported to increase lysosomal hydrolase synthesis in lung cancer cells, which is packaged into lysosomes and enlarge lysosomes (11). A hyperinvasive microenvironment is created as lysosomes secrete their protease cargo into the extracellular matrix under conditions of calcium flux (18–20).

We identified 14 genes out of 61 lysosome-related genes (LRGs) as significant predictors in LIHC. The 14 genes are ANKRD27, AP3M1, BCL10, CD63, CTSC, GLA, HPS1, HPS4, NPC2, PPT1, RAB7A, TPP1, VPS45, and RAMP3. The 14 lysosome-related genes were screened to classify molecular subgroups by NMF consensus clustering. The patients of LIHC from TCGA data were divided into C1 and C2 clusters. In addition, we explored that the most common CNVs are heterozygous amplifications and deletions of genes based on the LRGs. Further analysis of the pathway prediction and methylation was performed. An OS prognostic signature was constructed using LASSO regression analysis based on the LRGs. As an external verification database, we used the ICGC-LIHC cohorts for training the prognosis model.

We further explored the association between LRGs, immune checkpoints, and immune cells. Moreover, the TIDE scores of group G1 were significantly higher than those of group G2. The chemotherapy response in the two subtypes was also explored by comparing the IC50s of doxorubicin and cisplatin. We developed the nomogram by the univariate Cox regression model. Our result demonstrated that HPS4 and a worse prognosis are strongly associated with LIHC. We further explored the relation between HPS4 and immune cells based on eight single-cell datasets. We found that HPS4 could reduce proliferation and apoptosis significantly in liver cancer cells; it may be a promising therapeutic target and biomarker for LIHC, and regulating its activity may be an effective treatment strategy.

## 2 Materials and methods

### 2.1 Differential expression analysis of LRGs

We obtained 61 LRGs from the MSigDB (**Supplementary Table 1**). Expression profiles and clinical information for LIHC are provided via TCGA dataset (**Supplementary Table 2**). The raw read counts of genes were converted to transcripts per million (TPM) using STAR. Further analysis of LRG expression in LIHC was carried out using version R 3.6.3 and ggplot2. Our statistical significance criterion of log2(fold change) > 2 and adjusted p-value <0.05 indicated a significant difference.

### 2.2 Nomogram construction

Our goal was to identify the right terms for the nomogram by using univariate Cox regression analysis. For each variable, we calculated p values, HRs, and 95% confidence intervals using the “forestplot” R package. In order to predict 1-, 3-, and 5-year overall recurrence, a nomogram was developed using univariate Cox proportional hazards analysis. An individual’s recurrence risk can be calculated using nomograms by the “rms” R package.

### 2.3 The association between LRGs and gene mutation, CNV

Data about somatic mutations were provided by the UCSC Xena server for the GDC TCGA-LIHC project. According to the mutation order, the result was generated using the R package “maftools”. Data from TCGA database were downloaded and processed using GISTIC2.0, which can identify regions significantly altered including amplification and deletion.

### 2.4 The analysis of correlation between LRGs and immune infiltration

A TCGA dataset was downloaded to obtain LIHC clinical information and RNA sequencing profiles (level 3). LRG gene expression was correlated with immune scores using R’s ggstatsplot package, and multigene correlations were analyzed using R’s pheatmap package. To describe correlations between quantitative variables that do not follow a normal distribution, Spearman’s correlation analysis was used. R was used to implement all the analysis methods, and a statistical significance level below 0.05 was considered statistically significant.

### 2.5 Construct prognostic signature of LRGs

A TCGA dataset was downloaded to obtain LIHC clinical information and RNA sequencing profiles (level 3). In addition to converting count data to TPM, log2 (TPM+1) is normalized; it is important to keep samples together that contain information about the patient’s clinical status. A log-rank test was used to compare survival rates between the two groups. TimeROC (v0.4) was used to compare predictive accuracy between LRGs and risk scores. The features were selected using LASSO regression, cross-validated 10 times, and analyzed with the R package glmnet. With the survival package and Cox regression analysis, a prognostic model was constructed. We generated Kaplan–Meier curves with log-rank p-values and 95% confidence intervals (CIs) using log-rank tests. R was used to implement all the analysis methods, and a statistical significance level below 0.05 was considered statistically significant.

### 2.6 Identification of potential subtypes

80% of the samples were analyzed 100 times by ConsensusClusterPlus R package (v1.54.0). Using the R software package pheatmap (v1.0.12), we generated cluster heatmaps. Heatmaps showing gene expression were retained for genes with SD over 0.1. When the input gene number exceeded 1,000 after sorting the SD, the top 25% were extracted. All analysis methods and R packages were implemented in R version 4.0.3. 80% of TCGA LIHC samples (n = 371) were used as a discovery cohort to identify molecular subgroups. The remaining 20% of samples (n = 93) were held out as an internal validation set, to evaluate the reproducibility of the subgroups. Consensus clustering was run on the 80% discovery cohort, identifying two stable subgroups. These two subgroups were confirmed by testing their ability to classify the held-out 20% of samples.

### 2.7 The correlation between subtypes and immune

Evaluation of immune scores was conducted using immunedeconv. We extracted the expression of immune checkpoints including HAVCR2, PDCD1, CTLA4, LAG3 SIGLEC15, TIGIT, CD274, and PDCD1LG2. We predicted potential ICB responses using the TIDE algorithm and calculated mRNAsi based on the OCLR algorithm (21). Based on the mRNA expression signature, 11,774 genes are present in the gene expression profile. In order to map the dryness index to the range, the minimum value was subtracted and divided by the maximum value. R was used to implement all the analysis methods, and a statistical significance level below 0.05 was considered statistically significant.

### 2.8 The association of LRGs and drug sensitivity

With the help of the Genomics of Drug Sensitivity in Cancer (GDSC), we were able to predict chemotherapeutic response based on each sample. A prediction process was implemented using R package “pRRophetic”. The half-maximal inhibitory concentration (IC50) of samples was calculated using ridge regression. Using the batch effect of battle and tissue type, the duplicate gene expression values were summed up as a mean. R was used to implement all the analysis methods, and a statistical significance level below 0.05 was considered statistically significant.

### 2.9 Cell culture and transfection

From Procell (Wuhan, China), SK-hep-1 and Huh7 cells were obtained without mycoplasma infection. At 37°C with 5% CO_2_, the SK-hep-1 and Huh7 cell lines were cultured in MEM (Gibco, Grand Island, NY) and DMEM (Gibco), respectively. The medium includes 10% fetal bovine serum (FBS; Gibco), 100 U/mL penicillin, and 100 mg/mL streptomycin (Invitrogen, Waltham, MA). We transfected si-HPS4 and si-NC using Lipofectamine 2000 (Invitrogen). The cells were harvested 24 h after transfection.

### 2.10 Cell proliferation and apoptosis

Cells transfected with si-HPS4 were seeded in cell culture plates, 5,000 cells per well. A colony formation assay was performed by inoculating 500 cells into six-well plates and growing them at 37°C for 15 days. Following fixing the colonies with methanol and staining them with hematoxylin, TRIzol (Invitrogen) was used to extract RNA from liver cancer cells. All of the primer sequences and small interfering RNA sequences are listed in **Supplementary Table 2**. A 48- h transfection of si-HPS4 or si-NC was followed by harvesting of SK-hep-1 and Huh7 cells. The cells were then stained with FITC Annexin V Apoptosis Detection Kit (Meilunbio, Dalian, China) and detected using flow cytometry (Beckman Coulter, Brea, CA). We analyzed the results using the FCAP Array software. A triplicate of each trial was conducted.

### 2.11 Single-cell RNA sequencing-based analysis

We further explored the correlation between HPS4 and immune cells based on eight single-cell datasets by the TISCH2 resource (22). We next analyzed the expression of HPS4 in the hepatocyte population and hepatocellular carcinoma cells by single-cell and HCL resources (23). We further explored the gene expression levels in all cancer single-cell samples by the CancerSCEM resource (24).

## 3 Results

### 3.1 Identification of prognostic molecular subgroups

**Figure 1:**
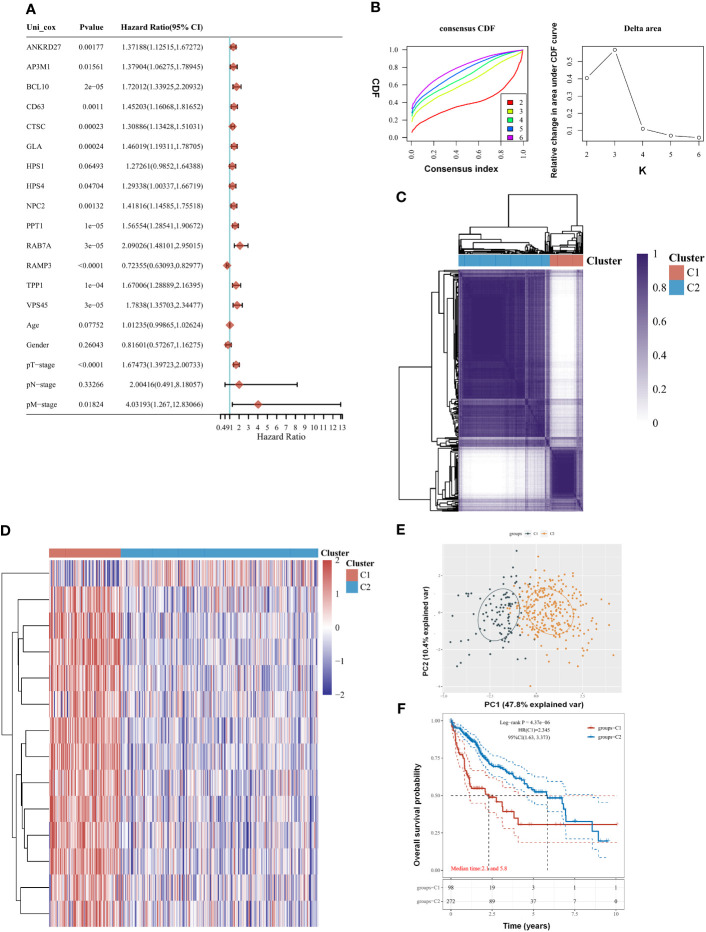
Clustering of molecular subgroup based on the lysosome-related genes (LRGs) in liver hepatocellular carcinoma (LIHC). **(A)** There were 14 genes out of 61 LRGs identified as significant predictors in LIHC. **(B)** K = 2 appeared to be the best clustering value for 14 genes by cumulative distribution function and function delta area. **(C)** There were 14 lysosome-related genes screened to classify molecular subgroups by NMF consensus clustering. **(D)** Heat maps of differentially expressed genes between the C1 and C2 clusters. **(E)** Principal component analysis (PCA). **(F)** Cluster 2 had a worse clinical prognosis compared with cluster 1. Statistical analyses were conducted by one-way ANOVA, principal component analysis, and log-rank test. p < 0.05 was considered statistically significant.

There were 14 genes out of 61 lysosome-related genes (LRGs) identified as significant predictors in LIHC. Among them, 13 are risk factors, namely, ANKRD27, AP3M1, BCL10, CD63, CTSC, GLA, HPS1, HPS4, NPC2, PPT1, RAB7A, TPP1, and VPS45, whereas RAMP3 is a favorable factor (**Figure 1A**). According to their cumulative distribution function and function delta area, k = 2 appeared to be the best clustering value for the 14 genes (**Figure 1B**). Initially, the 14 lysosome-related genes were screened to classify molecular subgroups by NMF consensus clustering. The patients of LIHC from TCGA data were divided into C1 and C2 clusters according to the consensus map (**Figure 1C**). Heat maps of differentially expressed genes show red for high expression and blue for low expression (**Figure 1D**). An additional confirmation was obtained by performing a principal component analysis (PCA) (**Figure 1E**). The log-rank test was used to examine the survival of the different groups by utilizing Kaplan–Meier curves (p < 0.0001) (**Figure 1F**). We found that cluster 2 had a worse clinical prognosis compared with cluster 1. Our result demonstrated that LRGs were associated with drug sensitivity based on the CTRP and GDSC resources (**Supplementary Figure 1**). The differential genes between cluster G1 and G2 were further explored (**Supplementary Figure 3**).

### 3.2 Analysis of LRG copy number variations and immune correlations

**Figure 2:**
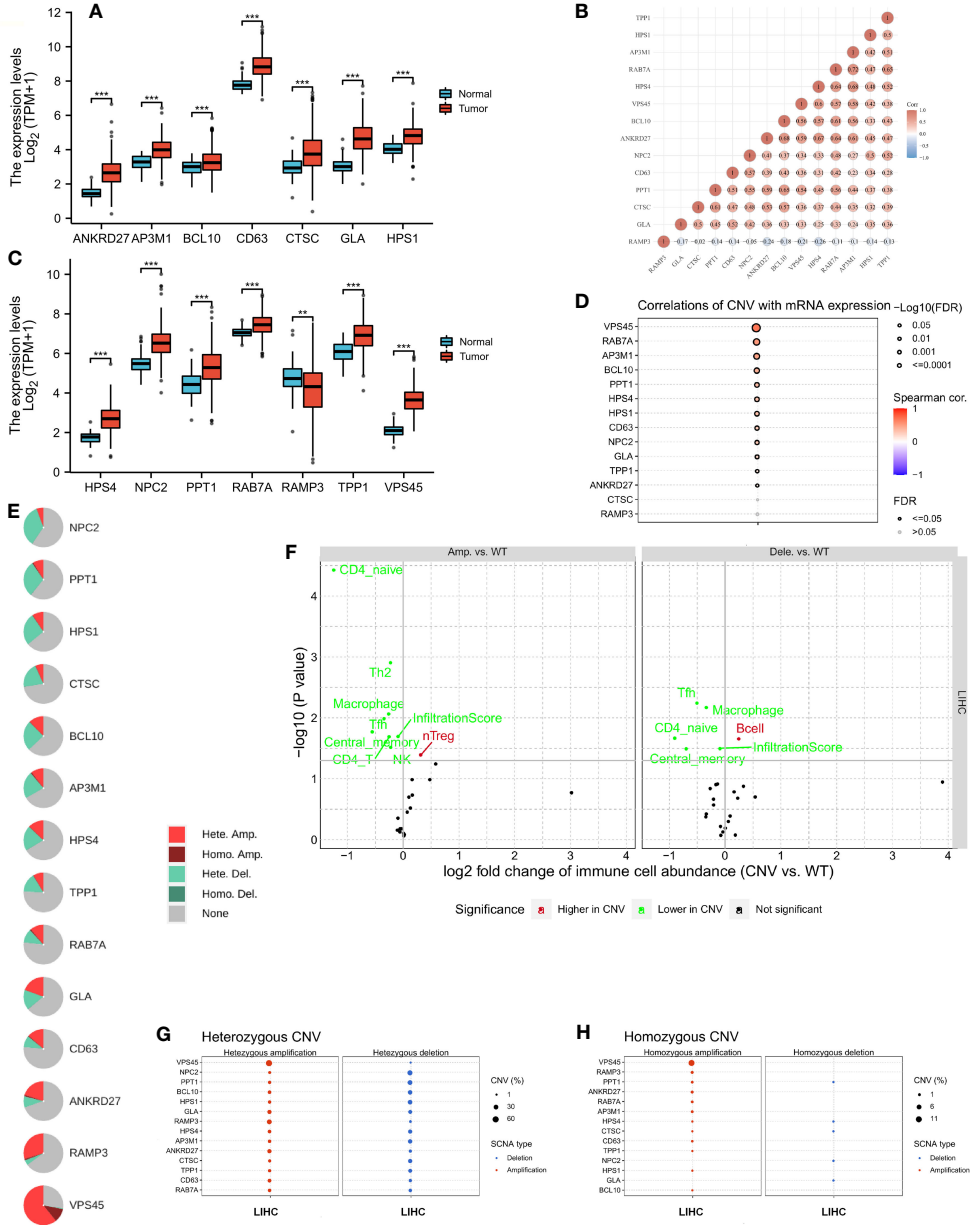
The copy number variation (CNV) of LRGs was correlated with immune cells. **(A, C)** A comparison of LRG expression between normal and tumor tissues was performed in LIHC. **(B)** A strong correlation was observed according to the expression of LRGs. **(D)** There was a significant positive correlation between CNV and most LRGs. **(E, G, H)** The most common CNVs are heterozygous amplifications and deletions of genes, whereas homozygous amplifications and deletions are much less common based on the LRGs’ **(F)** The relationship between immune and gene set CNV level was estimated. Statistical analyses were performed using t-test, Pearson correlation, and Fisher’s exact test. p < 0.05 was considered statistically significant. **p < 0.01; ***p < 0.001.

A comparison of LRG expression between normal and tumor tissues was performed in LIHC. The LRG expression in tumors was obviously higher than in normal tissues, whereas RAMP3 expression was significantly lower in tumors (**Figures 2A, C**). A strong correlation was observed according to LRG expression, such as RAB7A and AP3M1 which are most correlated (r = 0.72) (**Figure 2B**). There was a significant positive correlation between CNV and mRNA expression in most LRGs (**Figure 2D**). The copy number variation (CNV) is prevalent in human cancers, contributing to tumor development. We found that the most common CNVs are heterozygous amplifications and deletions of genes, whereas homozygous amplifications and deletions are much less common (**Figures 2E, G, H)**. The impact of tumor CNV levels on immune evasion is particularly intriguing. Analyzing the differences in immune infiltration between gene-set CNV groups, we estimate the relationship between immune and gene- set CNV levels. In the amplification group, NK cells, CD4 T cells, Tfh cells, Th2 cells, CD4 naïve cells, and macrophage, their infiltration scores all showed a negative correlation with CNV, whereas that of Treg cells was positively correlated with CNV in the group of amplification. The macrophage, Tfh cell, and CD4 naïve cell infiltration scores were negatively correlated with CNV in the group of deletion; however, the B- cell score showed the opposite trend (**Figure 2F**).

### 3.3 Pathway enrichment analysis and DNA methylation of LRGs

**Figure 3:**
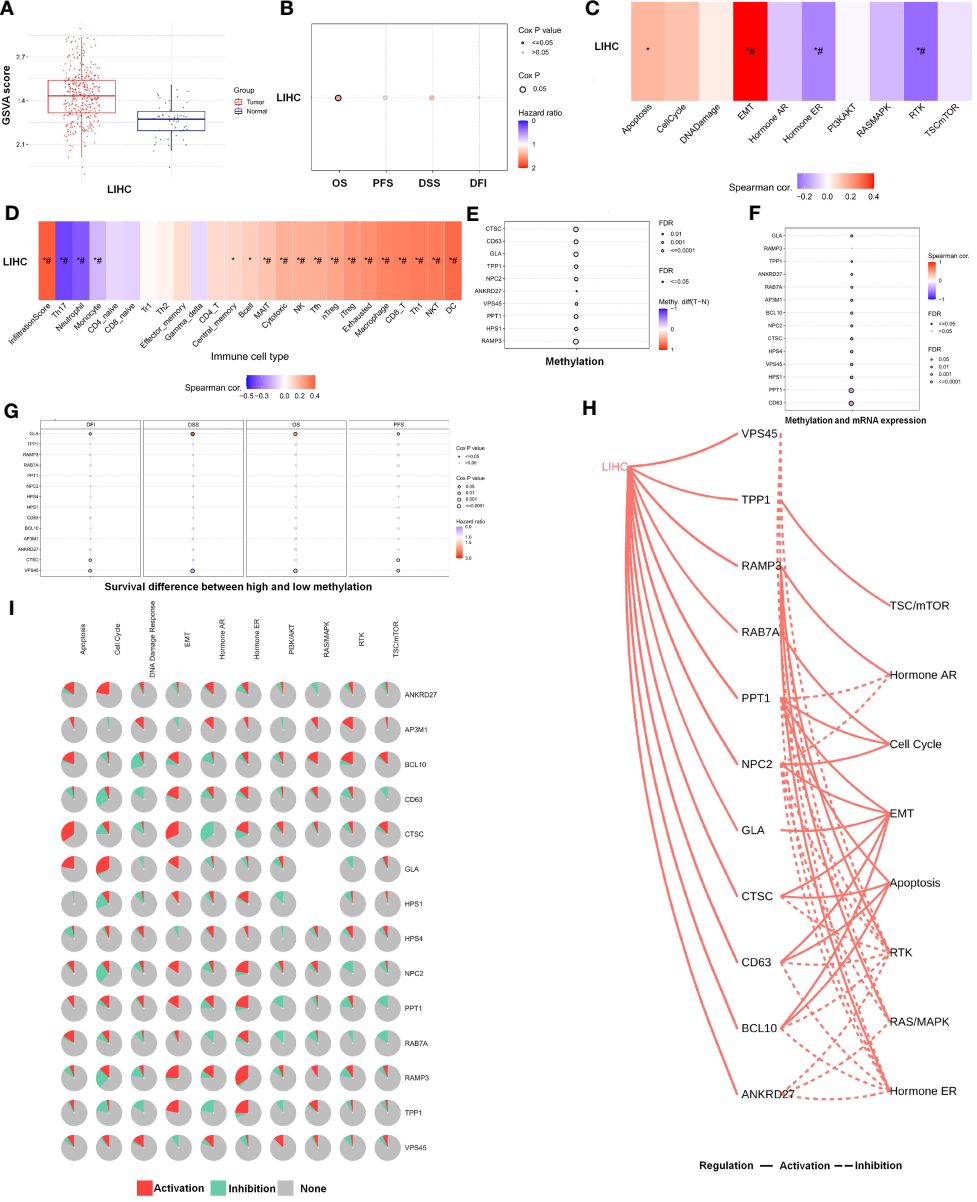
The analysis of pathway prediction and methylation. **(A)** The activity of differential gene sets between tumor and normal samples is analyzed based on the GSVA (gene set variation analysis) scores. **(B)** There was a worse prognosis in LIHC for patients with a high GSVA score. **(C)** The correlation between tumor-related pathways in LIHC and GSVA score was analyzed. **(D)** LRGs were associated with immune cell infiltrates using Spearman’s correlation analysis. **(E)** The hypomethylation level was explored based on the LRGs. **(F)** The association between methylation and mRNA expression was investigated. **(G)** The hypomethylation level of LRGs was associated with clinical prognosis. **(H, I)** The LRGs had a potential impact on pathway. Statistical analyses included t-test, Pearson correlation, log-rank test, and enrichment analyses. p < 0.05 was considered statistically significant. *p < 0.05; #FDR ≤ 0.05.

**Figure 4:**
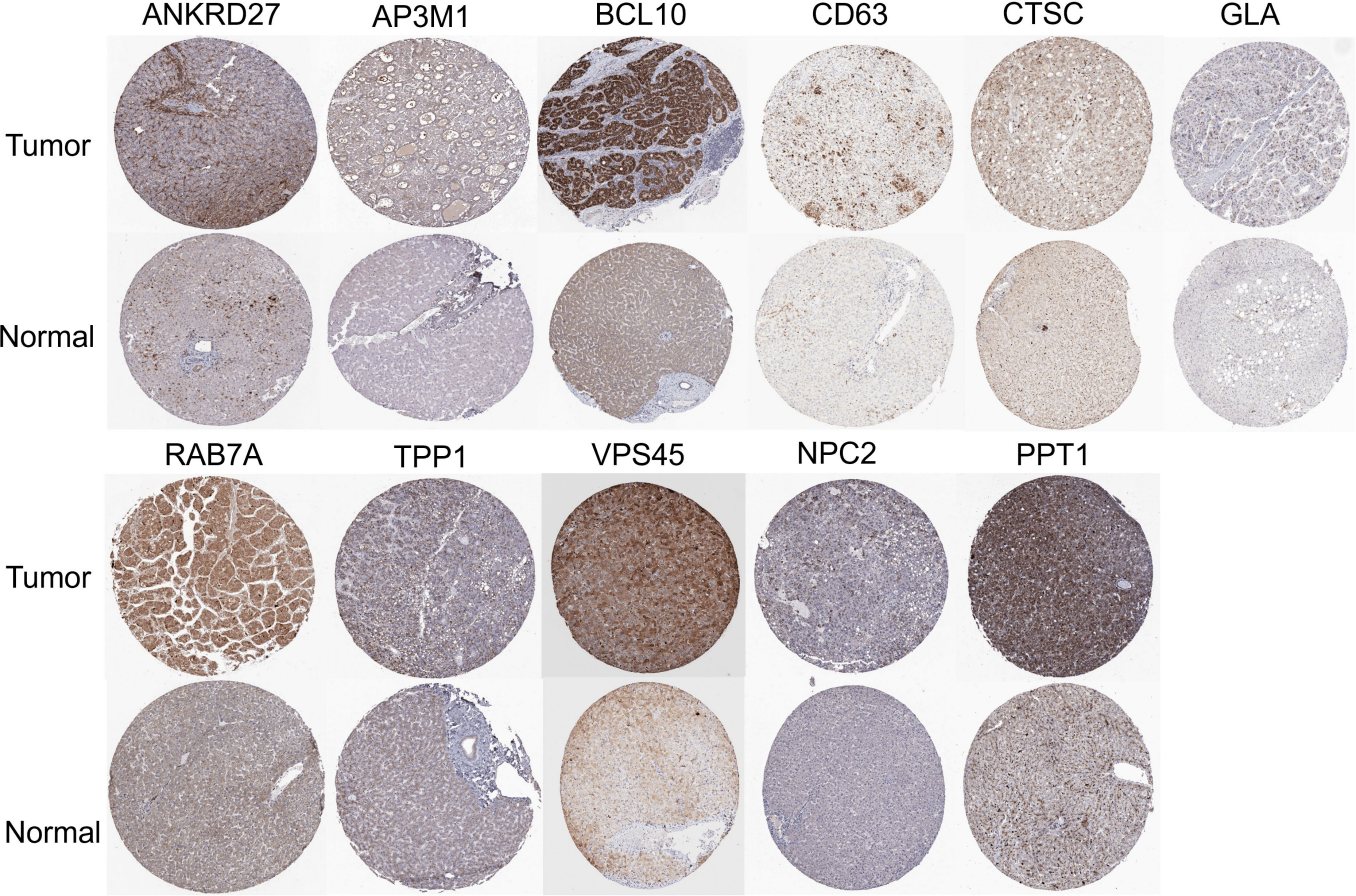
Immunohistochemistry staining images from the HPA resource were analyzed to further explore the LRGs protein expression in LIHC.

Further analysis of the CRGs’ GSVA (gene set variation analysis) scores was performed. The GSVA is an unsupervised method for measuring variations in gene- set activity (represented by GSVA scores) across cancer populations. By GSVA, the activity of differential gene sets between tumor and normal samples is analyzed. We found that the GSVA score was significantly increased in LIHC (**Figure 3A**). There was a worse prognosis in LIHC for patients with a high GSVA score (**Figure 3B**). In addition to the GSVA score, we studied the correlation between tumor-related pathways in the LIHC and the GSVA score. Our results demonstrated that GSVA score was positively associated with apoptosis and EMT pathway, and GSVA score was negatively associated with hormone ER and RTK pathway (**Figure 3C**). Additionally, LRG expression was associated with immune cell infiltrates using Spearman’s correlation analysis. We found that LRGs were negatively associated with Th17 cell, neutrophil, and monocyte, whereas LRGs were positively associated with cytotoxic cell, NK cell, Tfh cell, Treg cell, macrophage, CD8 T cell, Th1 cell, NKT cell, and DC cell (**Figure 3D**). In addition, we found that PPT1, HPS1, and RAMP3 were hypermethylated and CTSC, CD63, and GLA have a hypomethylation level (**Figure 3E**). Methylation and mRNA expressions were also investigated. We explored that most of LRGs had a negative association with methylation including ANKRD27, RAB7A, AP3M1, BCL10, NPC2, CTSC, HPS4, VPS45, HPS1, PPT1, and CD63 whereas GLA had a positive association with methylation (**Figure 3F**). In the LIHC cohorts, hypomethylation of VPS45 had an association with a worse prognosis whereas hypermethylation of GLA showed the opposite situation (**Figure 3G**). Our results demonstrated that LRGs had potential impact on pathways including the TSC/mTOR, RTK, RAS/MAPK, PI3K/AKT, hormone ER, hormone AR, EMT, DNA damage response, cell cycle, and apoptosis pathways. Most LRGs could activate the apoptotic cell cycle and the EMT pathway, inhibiting the RTK and TSC/mTOR pathways (**Figures 3I, H**). Immunohistochemistry staining images from the HPA were analyzed to further explore the LRG protein expression in LIHC. Our results demonstrated that the expression of most LRG proteins was higher in tumor tissues (**Figure 4**).

### 3.4 Development of an LRG-based prognostic signature model

**Figure 5:**
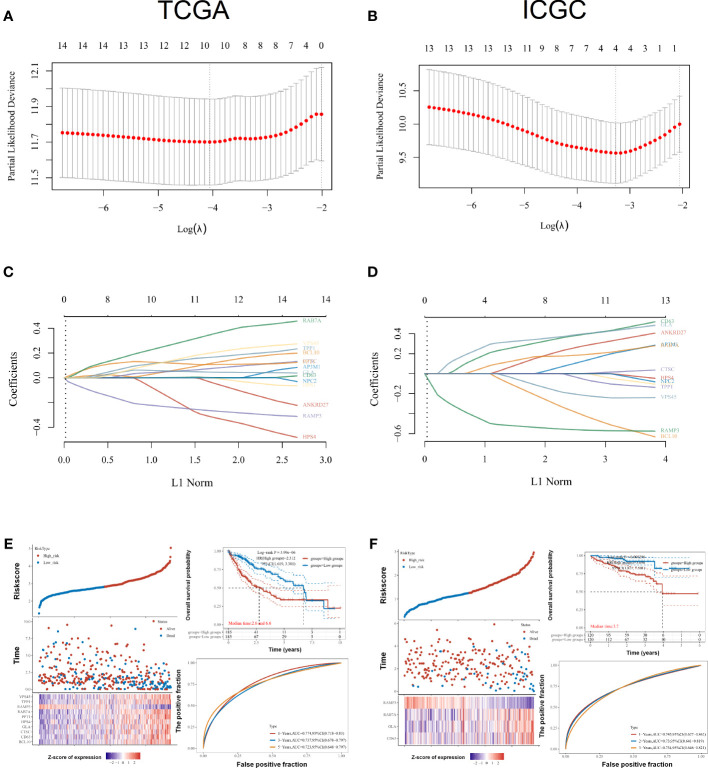
Prognostic signature construction for LRGs. **(A, C)** Based on univariate Cox regression, LRGs were selected for LASSO regression. **(B, D)** As an external verification database, we selected the ICGC-LIHC cohorts for training the prognosis model. **(E)** Survival of LIHC patients was significantly associated with LRG-related risk signatures at the individual level in the TCGA-LIHC cohorts. **(F)** There was a significant association between LRGs’ signature risk score and poor overall survival in the ICGC-LIHC cohorts. Statistical analyses utilized LASSO regression, log-rank test, and time-dependent ROC analysis. p < 0.05 was considered statistically significant.

As part of this research, we evaluated the relationship between LRGs and the prognosis of LIHC patients. Based on univariate Cox regression, LRGs were selected for LASSO regression (**Figures 5A, C**). An OS prognostic signature was constructed using LASSO regression analysis for 10 genes of LRGs: risk score = (0.0817)*BCL10+(0.0043)*CD63+(0.0601)*CTSC+(0.0502)*GLA+(−0.2469)*HPS4+(0.1112)*PPT1+(0.3027)*RAB7A+(-0.2508)*RAMP3+(0.1498)*TPP1+(0.1685)*VPS45 (lambda.min = 0.0173). There was a significant association between LRGs’ signature risk score and poor overall survival in the study (HR = 2.312 (1.619–3.301), log-rank p = 3.99e−06). In terms of AUCs, the 1-, 3-, and 5-year ROC curves were accurate by 0.774, 0.737, and 0.723, respectively. Survival of LIHC patients was significantly associated with LRG-related risk signatures at the individual level (**Figure 5E** **)**. As an external verification database, we used the ICGC-LIHC cohorts for training the prognosis model. Based on univariate Cox regression, LRGs were selected for LASSO regression in the ICGC-LIHC cohorts **(** **Figures 5B, D** **)**. There was a significant association between LRGs’ signature risk score and poor overall survival in the ICGC-LIHC cohorts (HR = 3.696 (1.821–7.501), log-rank p = 0.000296, **Figure 5F**). The association between subtypes and ferroptosis and m6A was further performed (**Supplementary Figure 2**). We also found that the relative expression levels of macrophages were lower in the high-risk group (**Supplementary Figure 4**).

### 3.5 Evaluation of subtype-specific immune landscape and drug response

**Figure 6:**
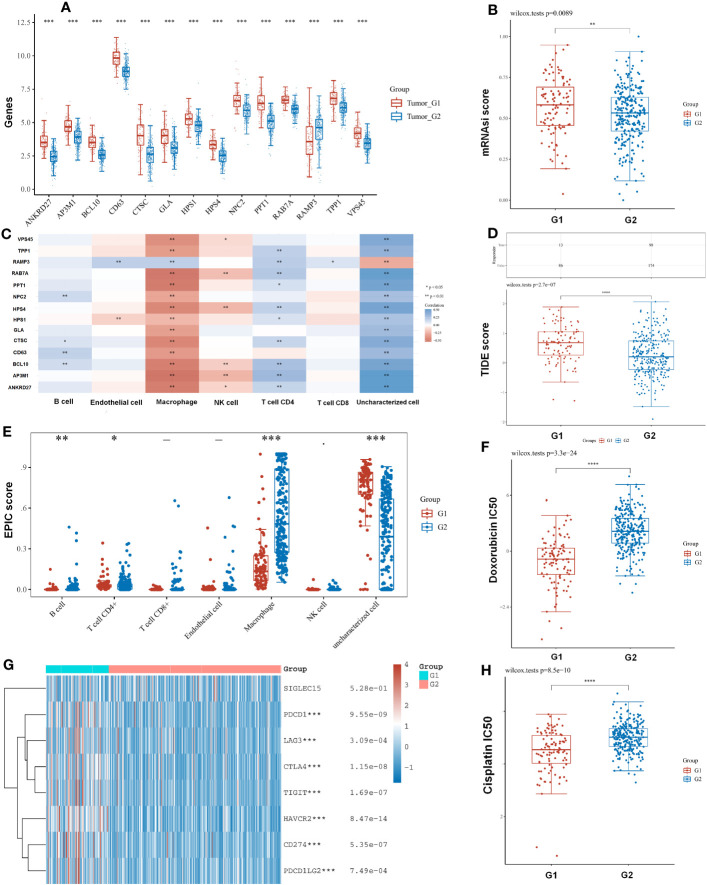
The subtypes had a correlation with immune and immune response. **(A)** A comparison of LRG expression in the two subtypes was also carried out. **(B)** With a better clinical prognosis, the G2 group had lower mRNAsi scores. **(C)** LRGs were associated with immune infiltration by the EPIC algorithm. **(D)** TIDE scores of group G1 were significantly higher than those of group G2. **(E)** The subtypes had a significant association with immune cell. **(G)** The G1 group having a greater abundance of immune checkpoints than the G2 group. **(F, H)** The chemotherapy response in the two subtypes was further explored by comparing the IC_50_s of doxorubicin and cisplatin. Statistical analyses were conducted by t-test, Pearson correlation, log-rank test, and ANOVA. p < 0.05 was considered statistically significant. *p < 0.05; **p < 0.01; ***p < 0.001.

A comparison of LRG expression in the two subtypes was also carried out. We found that most LRGs increased significantly in group G1 whereas gene RAMP3 showed the opposite trend (**Figure 6A**). In tumors with higher mRNAsi, the tumor has dedifferentiated more and its cancer stem cells are more active. With a better clinical prognosis, the G2 group had lower mRNAsi scores whereas the G1 group had higher mRNAsi scores (**Figure 6B**). Our results demonstrated that ANKRD27, AP3M1, BCL10, CTSC, HPS1, HPS4, PPT1, RAB7A, TPP1, and RAMP3 had a negative relation to immune infiltration of CD4 T cells using the EPIC algorithm. Moreover, ANKRD27, AP3M1, BCL10, CD63, CTSC, GLA, HPS1, HPS4, NPC2, PPT1, RAB7A, TPP1, and VPS45 had a positive correlation with macrophage (**Figure 6C**). Furthermore, the TIDE algorithm showed low efficacy of immune checkpoint blockade therapy (ICB) based on high TIDE scores. As a result, the TIDE scores of group G1 were significantly higher than those of group G2 (**Figure 6D**). We found that subtypes had a significant association with immune cells including B cell, CD4 T cell, CD8 T cell, endothelial cell, macrophage, and NK cell. Macrophages in the G1 group had lower immune scores than those in the G2 group (**Figure 6E**). Furthermore, the subtypes were significantly related to immune checkpoints, with the G1 group having a greater abundance of immune checkpoints than the G2 group (**Figure 6G**). The chemotherapy response in the two subtypes was further explored by comparing the IC_50_s of doxorubicin and cisplatin. A significant decrease in chemotherapy response was observed in G1 compared with G2 based on IC50 of doxorubicin and cisplatin (**Figures 6F, H**).

### 3.6 HPS4 as a potential therapeutic target in LIHC

**Figure 7:**
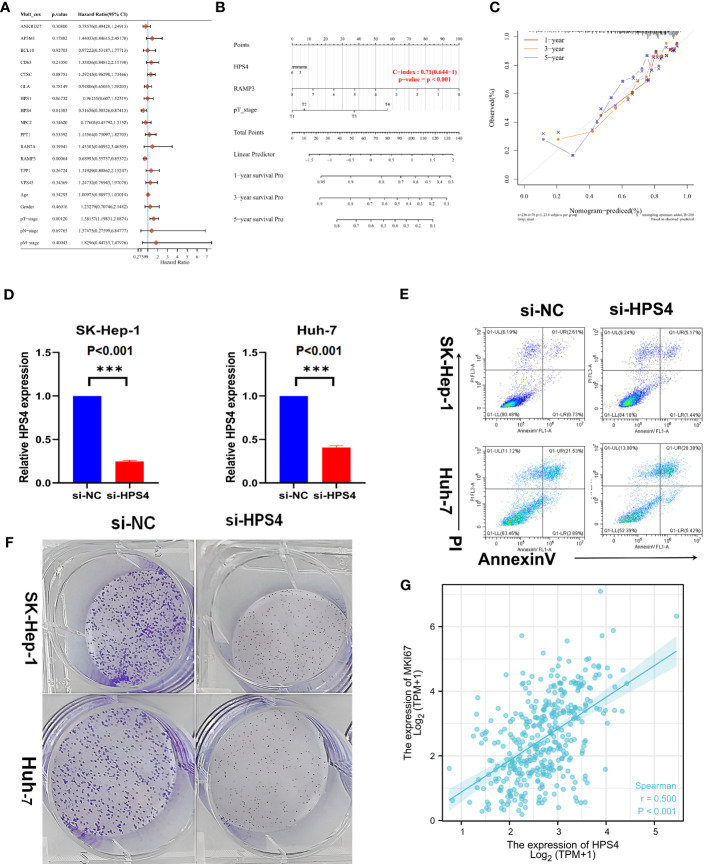
Identifying HPS4 as novel therapy target for LIHC. **(A)** A univariate Cox regression model was used to develop the nomogram. **(B)** The nomogram displayed relatively good predictive ability. **(C)** The predicted OS and observed OS at 1, 3, and 5 years. **(D)** SK-hep-1 and Huh7 cells were treated with si-HPS4 to interfere with HPS4 expression. **(E)** A significant increase in apoptotic cells was observed when HPS4 was knocked down by si-HPS4. **(F)** The interference of HPS4 with SK-hep-1 and Huh7 cells significantly decreased proliferation by the colony formation assay. **(G)** The association between HPS4 and a proliferation marker (MKI67). Statistical analyses included Cox regression, log-rank test, t-test, and Pearson correlation. p < 0.05 was considered statistically significant. ***p < 0.001.

A univariate Cox regression model was used to develop the nomogram (**Figure 7A**). On the basis of its c-index of 0.71, it displayed relatively good predictive ability (**Figure 7B**). According to the calibration plots, the predicted OS and observed OS at 1, 3, and 5 years were well concordant (**Figure 7C**). As HPS4 and a worse prognosis are strongly associated with LIHC, we further explored whether HPS4 contributes to liver cancer proliferation. SK-hep-1 and Huh7 cells were treated with si-HPS4 to interfere with HPS4 expression (**Figure 7D**). Moreover, apoptosis could contribute to tumor cell growth; the level of apoptosis in liver cancer cells was also detected using flow cytometry. A significant increase in apoptotic cells was observed when HPS4 was knocked down by si-HPS4 (**Figure 7E**). The interference of HPS4 with SK-hep-1 and Huh7 cells significantly decreased proliferation by the colony formation assay (**Figures 7F**). Furthermore, we analyzed the association between HPS4 and a proliferation marker (MKI67). We found that HPS4 was positively related to MKI67 (**Figures 7G**). Collectively, HPS4 may be a promising therapeutic target and biomarker for LIHC, and regulating its activity may be an effective treatment strategy.

### 3.7 Analysis of single-cell RNA sequencing data

**Figure 8:**
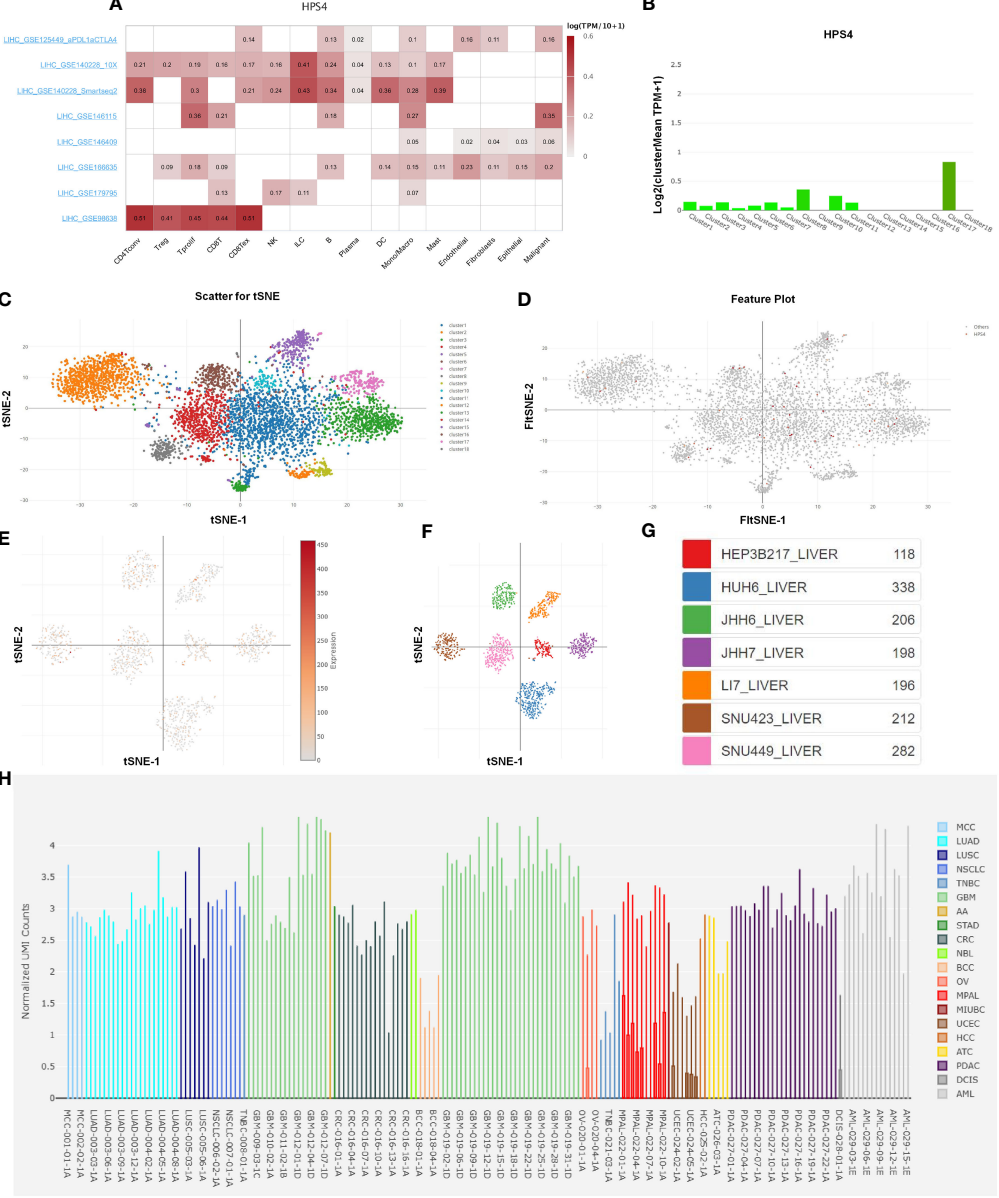
The analysis of single-cell RNA sequencing. **(A)** HPS4 had a significant correlation with CD8 T, B cell, and macrophage based on eight single-cell datasets. **(B–D)** HPS4 was expressed in cluster 1, cluster 3, cluster 6, cluster 8, cluster 10, cluster 11, and cluster 17. Their corresponding cell populations are activated T cell, sinusoidal endothelial cell, myeloid cell, dendritic cell, smooth muscle cell, hepatocyte_APOA2 high, and epithelial cell_SCGB3A1 high. **(E–G)** HPS4 was also expressed in hepatocellular carcinoma cells including HEP3B217, HUH6, JHH6, JHH7, LI7, SNU423, and SNU449. **(H)** The HPS4 expression level was explored in all cancer single-cell samples. Correlation, clustering, and differential expression analyses were performed. p < 0.05 was considered statistically significant.

We further explored the relation between HPS4 and immune cells based on eight single-cell datasets. Our results demonstrated that HPS4 had a significant correlation with CD8 T cell, B cell, and macrophage (**Figure 8A**). We next analyzed the expression of HPS4 in the hepatocyte population. We found that HPS4 was expressed in cluster 1, cluster 3, cluster 6, cluster 8, cluster 10, cluster 11, and cluster 17. Their corresponding cell populations are activated T cell, sinusoidal endothelial cell, myeloid cell, dendritic cell, smooth muscle cell, hepatocyte_APOA2 high, and epithelial cell_SCGB3A1 high (**Figures 8B–D**). Similarly, we found that HPS4 was also expressed in hepatocellular carcinoma cells including HEP3B217, HUH6, JHH6, JHH7, LI7, SNU423, and SNU449 (**Figures 8E–G**). The gene expression level was explored in all cancer single-cell samples. HPS4 was highly expressed in GBM, LUAD, PDAC, and AML relative to other tumors (**Figure 8H**).

## 4 Discussion

Globally, LIHC ranks sixth among the most common cancers. Despite the application of new treatment strategies for LIHC, efficacy remains unsatisfactory. Thus, identification of specific prognostic markers is essential for guiding LIHC therapy and improving OS of patients. It has been reported that lysosome functions are drastically altered during cancer progression, including alterations in lysosome volume, localization, and composition within the cell. A number of cancers show significant overexpression of lysosomal hydrolases, which increases invasion of the tumor (11). It is therefore imperative to understand how LRGs affect LIHC and determine if they serve as prognostic indicators. There has not been any reporting on a prognostic model based on LRGs.

To verify the efficacy of lysosome-related genes on the prognosis of hepatocellular carcinoma, we identified 14 genes out of 61 lysosome-related genes (LRGs) as significant predictors in LIHC. The 14 genes are ANKRD27, AP3M1, BCL10, CD63, CTSC, GLA, HPS1, HPS4, NPC2, PPT1, RAB7A, TPP1, VPS45, and RAMP3. The 14 lysosome-related genes were screened to classify molecular subgroups by NMF consensus clustering. The patients of LIHC from TCGA data were divided into C1 and C2 clusters. We found that cluster 2 had a worse clinical prognosis compared with cluster 1. In addition, we explored that most common CNVs are heterozygous amplifications and deletions of genes based on the LRGs. The impact of tumor CNV levels on immune evasion is particularly intriguing. We also analyzed the differences in immune infiltration between gene-set CNV groups. Further analysis of the pathway prediction and methylation was performed. An OS prognostic signature was constructed using LASSO regression analysis based on the LRGs. As an external verification database, we used the ICGC-LIHC cohorts for training the prognosis model.

Tumor-associated immune responses are regulated and determined by immune cells in the tumor microenvironment (25–27). We also explored the association between LRGs and immune cells. According to some studies, declining CD4+ T cell counts can contribute to liver cancer development (28, 29), whereas our results demonstrated that ANKRD27, AP3M1, BCL10, CTSC, HPS1, HPS4, PPT1, RAB7A, TPP1, and RAMP3 had a negative relation to immune infiltration of CD4 T cells using the EPIC algorithm. We found that subtypes had a significant association with immune cells including B cell, CD4 T cell, CD8 T cell, endothelial cell, macrophage, and NK cell. Several cancer types show a correlation between macrophage density and poor prognosis, suggesting that macrophages play a key role in tumor progression (30–32). Macrophages in the G1 group had lower immune scores than those in the G2 group. Furthermore, the TIDE algorithm showed low efficacy of immune checkpoint blockade therapy (ICB) based on high TIDE scores (21, 33). As a result, the TIDE scores of group G1 were significantly higher than those of group G2. A profound advance in cancer therapy has come from the discovery that immunocheckpoint molecules overexpress in the tumor microenvironment and contribute to antitumor immunity evasion (34–36). Furthermore, the subtypes were significantly related to immune checkpoints, with the G1 group having a greater abundance of immune checkpoints than the G2 group. The resistance to chemotherapy contributes significantly to cancer mortality (37, 38). Thus, the chemotherapy response in the two subtypes was further explored by comparing the IC_50_s of doxorubicin and cisplatin. A significant decrease in chemotherapy response was observed in G1 compared with G2 based on the IC_50_ of doxorubicin and cisplatin.

We developed the nomogram by the univariate Cox regression model. Our result demonstrated that HPS4 and a worse prognosis are strongly associated with LIHC. SK-hep-1 and Huh7 cells were treated with si-HPS4 to interfere with HPS4 expression. The interference of HPS4 with SK-hep-1 and Huh7 cells significantly decreased proliferation and colony formation. Moreover, apoptosis could contribute to tumor cell growth; the level of apoptosis in liver cancer cells was also detected using flow cytometry. A significant increase in apoptotic cells was observed when HPS4 was knocked down by si-HPS4. We found that HPS4 was positively related to MKI67 whereas MKI67 is an important marker of proliferation (39). As a tool for studying cell types and states, single-cell RNA sequencing (scRNA-seq) is becoming increasingly important. We further explored the relation between HPS4 and immune cells based on eight single-cell datasets. Our results demonstrated that HPS4 had a significant correlation with CD8 T, B cell, and macrophage. We found that the genes were mainly expressed in activated T cell, sinusoidal endothelial cell, myeloid cell, dendritic cell, smooth muscle cell, hepatocyte_APOA2 high, and epithelial cell_SCGB3A1 high. Collectively, it turns out that HPS4 may be a promising therapeutic target and biomarker for LIHC, and regulating its activity may be an effective treatment strategy.

The study has some limitations. There is still a lack of understanding of how HPS4 impacts proliferation or apoptosis of liver cancer cells. This is our next endeavor to further explore how HPS4 regulates LIHC *in vivo* and *in vitro*.

## 5 Conclusion

A prognostic model based on LRGs was developed in this study to predict the prognosis of patients with LIHC. HPS4, which could affect proliferation and apoptosis of liver cancer cells, may be a promising therapeutic target and biomarker for LIHC. HPS4 may be a promising therapeutic target and biomarker for LIHC, and regulating its activity may be an effective treatment strategy.

## Data availability statement

The original contributions presented in the study are included in the article/**Supplementary Material**. Further inquiries can be directed to the corresponding author.

## Author contributions

K-JH designed this work. K-JH performed bioinformatic analyses. K-JH wrote the manuscript. K-JH and ZN revised the manuscript and supervised the whole experiment. All authors have read and approved the final submitted manuscript.

## Supplementary material

Supplementary Figure 1 LRGs were associated with drug sensitivity based on the CTRP (a) and GDSC resource (b). Statistical analysis was performed using Pearson correlation. P < 0.05 was considered statistically significant. *P < 0.05; **P < 0.01; *** P < 0.001.

Supplementary Figure 2 The association between subtypes and ferroptosis and m6A. Enrichment analysis and correlation tests were conducted. P < 0.05 was considered statistically significant. *P < 0.05; **P < 0.01; *** P < 0.001.

Supplementary Figure 3 The differential genes between cluster G1 and G2; (a) The volcano plot; (b) heat map of differential genes; (c) Pathway enrichment analysis. P < 0.05 was considered statistically significant. *P < 0.05; **P < 0.01; *** P < 0.001.

Supplementary Figure 4 Correlation between high and low risk groups and immune cells. Correlation analysis was conducted using Pearson correlation. P < 0.05 was considered statistically significant. *P < 0.05; **P < 0.01; *** P < 0.001.
