# Supplementary figures and images for: System analysis based on the lysosome-related genes identifies HPS4 as a novel therapy target for liver hepatocellular carcinoma
Source: Front Oncol. 2023 Sep 13;13:1221498. doi: 10.3389/fonc.2023.1221498 (PMC10535104; doi:10.3389/fonc.2023.1221498)

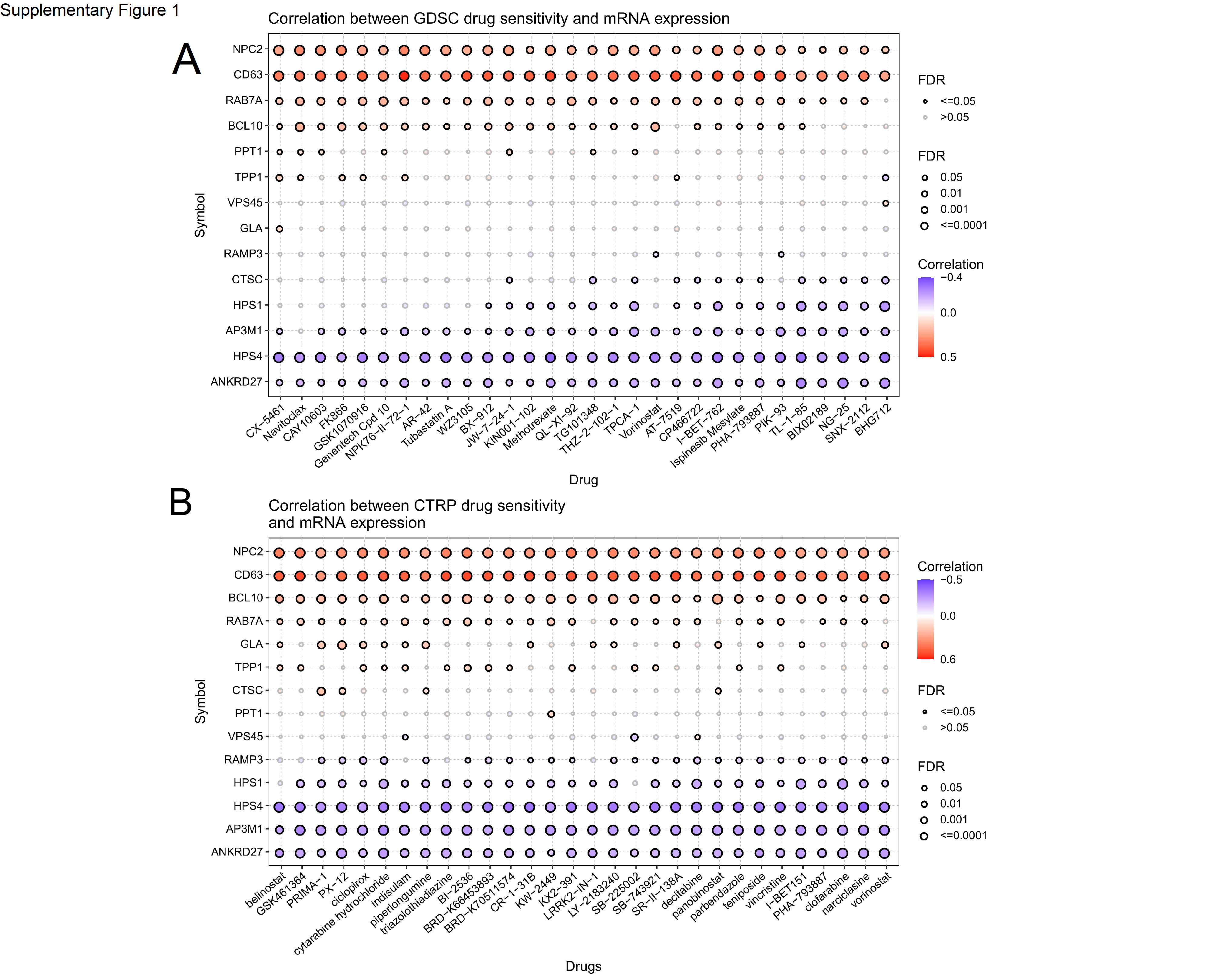

Supplement: Supplementary Figure 1 — LRGs were associated with drug sensitivity based on the CTRP (a) and GDSC resource (b). Statistical analysis was performed using Pearson correlation. P < 0.05 was considered statistically significant. *P < 0.05; **P < 0.01; *** P < 0.001. [file Image_1.tiff]

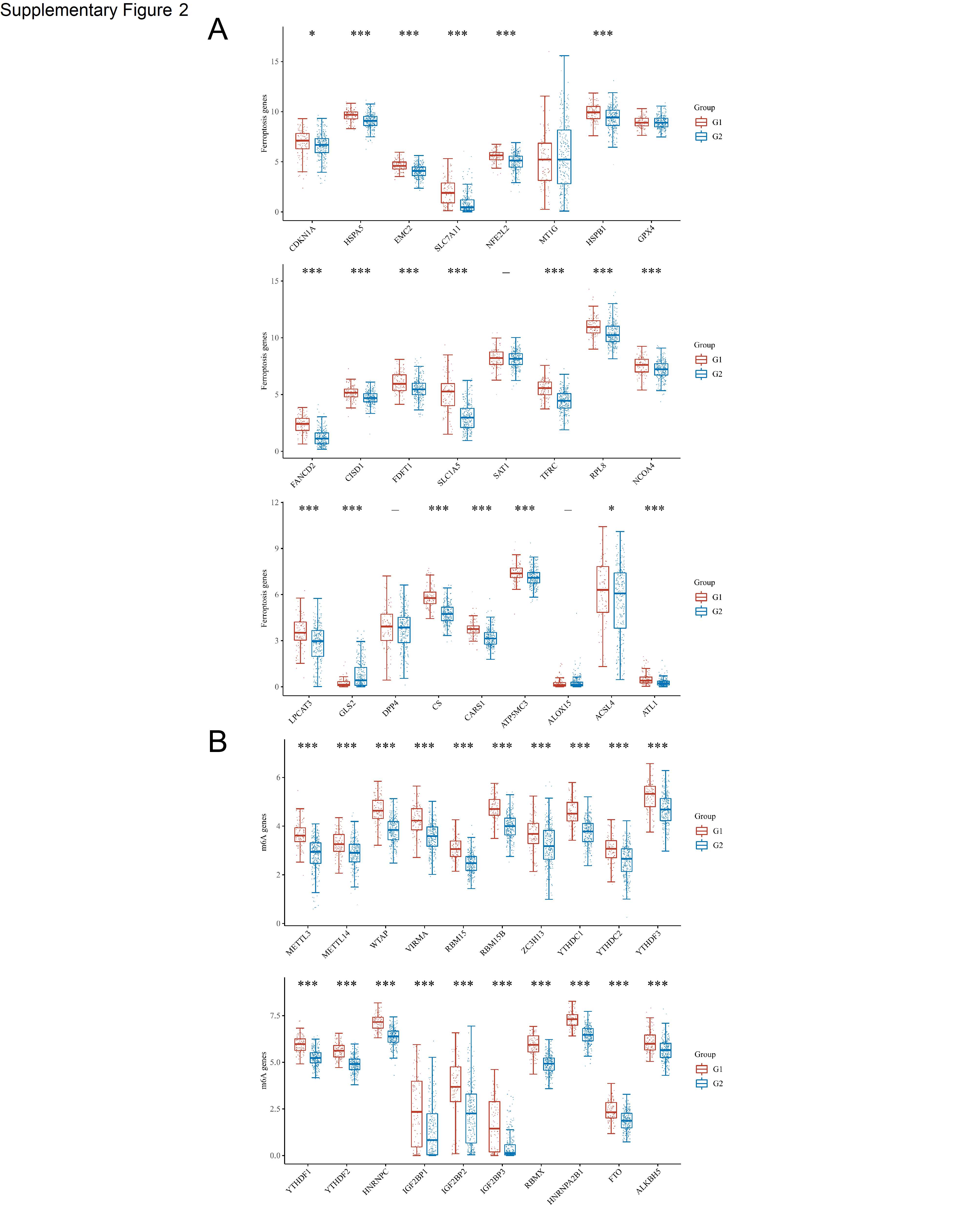

Supplement: Supplementary Figure 2 — The association between subtypes and ferroptosis and m6A. Enrichment analysis and correlation tests were conducted. P < 0.05 was considered statistically significant. *P < 0.05; **P < 0.01; *** P < 0.001. [file Image_2.tiff]

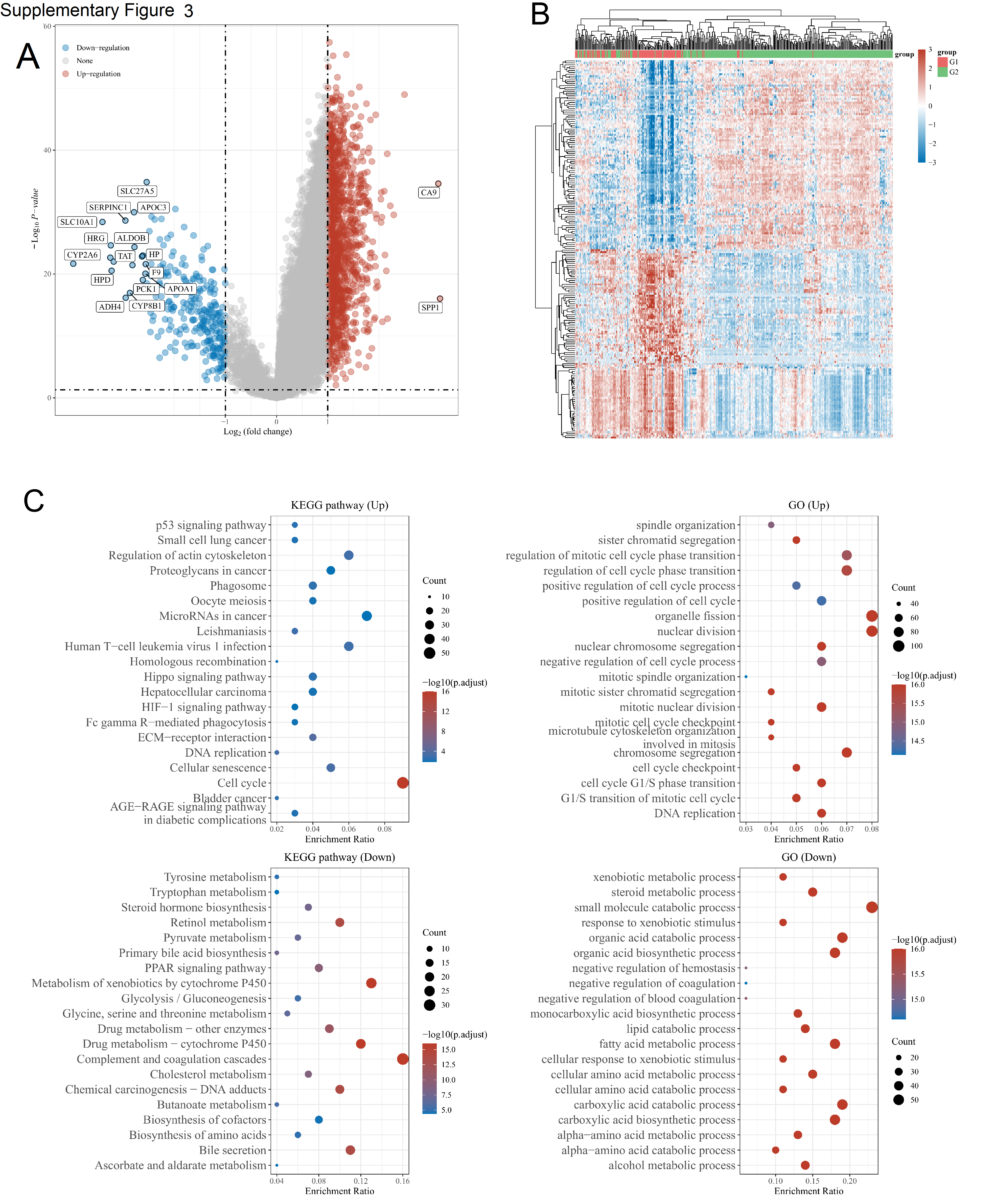

Supplement: Supplementary Figure 3 — The differential genes between cluster G1 and G2; (a) The volcano plot; (b) heat map of differential genes; (c) Pathway enrichment analysis. P < 0.05 was considered statistically significant. *P < 0.05; **P < 0.01; *** P < 0.001. [file Image_3.tiff]

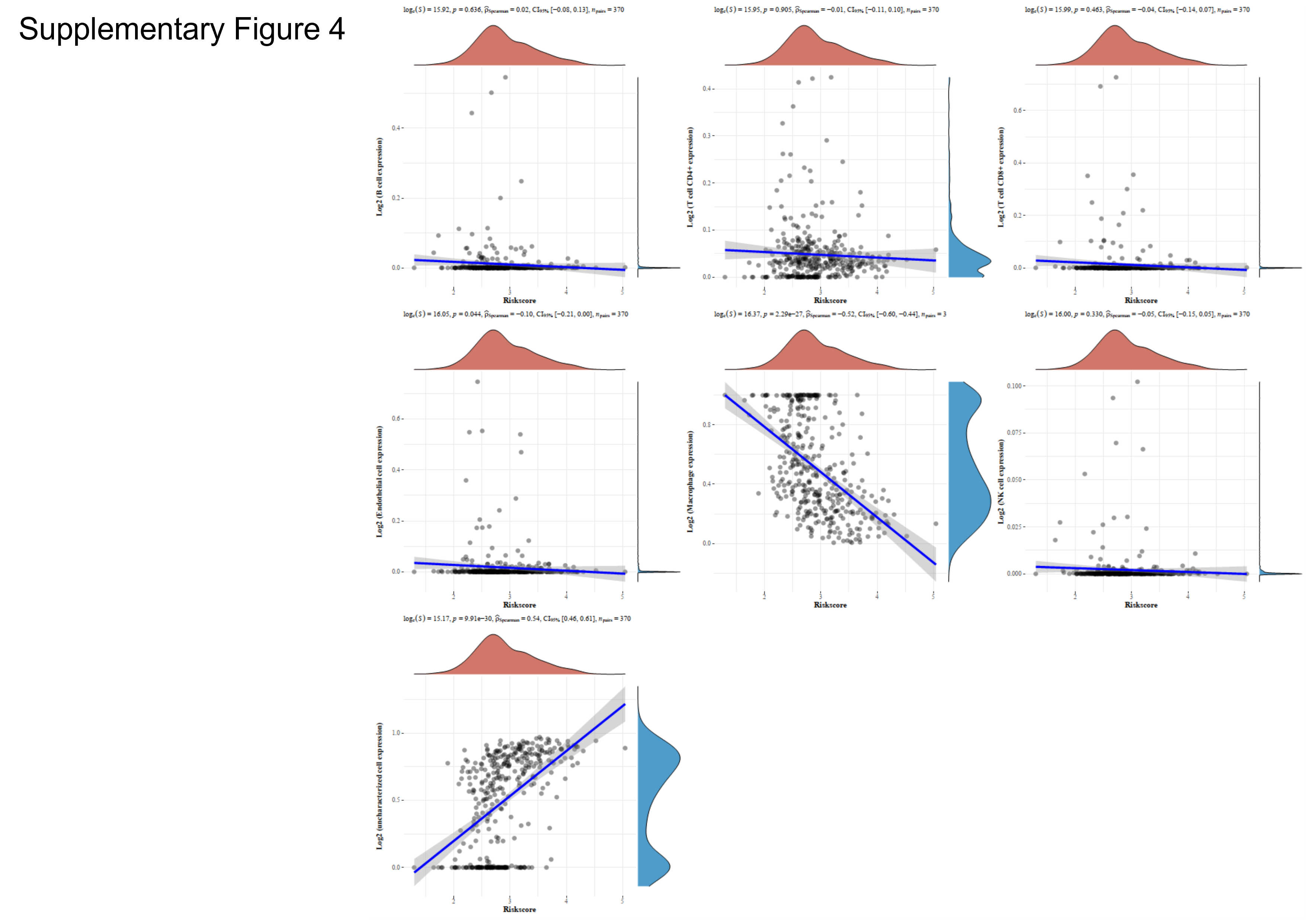

Supplement: Supplementary Figure 4 — Correlation between high and low risk groups and immune cells. Correlation analysis was conducted using Pearson correlation. P < 0.05 was considered statistically significant. *P < 0.05; **P < 0.01; *** P < 0.001. [file Image_4.tif]
