# Supplementary material for: System analysis based on the lysosome-related genes identifies HPS4 as a novel therapy target for liver hepatocellular carcinoma
Source: Front Oncol. 2023 Sep 13;13:1221498. doi: 10.3389/fonc.2023.1221498 (PMC10535104; doi:10.3389/fonc.2023.1221498)
Supplement: Supplementary file 6 [file Table_2.doc]

**qRT-PCR primers and siRNA sequences**

| **qRT-PCR primers** | | |
| --- | --- | --- |
| **Gene** | **Forward** | **Reverse** |
| HPS4 | GTGACCTAAGGCCTCTCTGC | GTTGGTCTAGCAGGGTCTGG |
| GAPDH | TGCACCACCAACTGCTTAGC | GGCATGGACTGTGGTCATGAG |
| **siRNA sequences** |  |  |
| si-HPS4 | CCTCAGTTCTATTACGTTATA |  |
